# ZIKV can infect human term placentas in the absence of maternal factors

**DOI:** 10.1038/s42003-022-03158-6

**Published:** 2022-03-18

**Authors:** Diana L. Villazana-Kretzer, Kathryn McGuckin Wuertz, Daniel Newhouse, Jennifer R. Damicis, Elisabeth M. Dornisch, Kathleen M. Voss, Antonio E. Muruato, Jennifer A. Paymaster, Stacey S. Schmiedecke, Sarah M. Edwards, Peter G. Napolitano, Jennifer Tisoncik-Go, Nicholas Ieronimakis, Michael Gale

**Affiliations:** 1 Division of Maternal Fetal Medicine, Madigan Army Medical Center, Tacoma, WA USA; 2 Center for Innate Immunity and Immune Disease, Department of Immunology, University of Washington School of Medicine, Seattle, WA USA; 3 Department of Clinical Investigation, Madigan Army Medical Center, Tacoma, WA USA; 4 Department of OB/GYN, University of Washington Medical Center, Seattle, WA USA

**Keywords:** Infection, Viral infection

## Abstract

Zika virus infection can result in devastating pregnancy outcomes when it crosses the placental barrier. For human pregnancies, the mechanisms of vertical transmission remain enigmatic. Utilizing a human placenta-cotyledon perfusion model, we examined Zika virus exposure in the absence of maternal factors. To distinguish responses related to viral infection *vs*. recognition, we evaluated cotyledons perfused with either active or inactivated Zika virus. Active Zika virus exposure resulted in infection, cell death and syncytium injury. Pathology corresponded with transcriptional changes related to inflammation and innate immunity. Inactive Zika virus exposure also led to syncytium injury and related changes in gene expression but not cell death. Our observations reveal pathologies and innate immune responses that are dependent on infection or virus placenta interactions independent of productive infection. Importantly, our findings indicate that Zika virus can infect and compromise placentas in the absence of maternal humoral factors that may be protective.

## Abstract (web summary)

Villazana-Kretzer et al. compare histology, physiology and gene expression in cotyledons from term placentas perfused with either active or UV-inactivated Zika virus. They show that ZIKV can infect human term placentas in the absence of maternal factors and identify unique transcriptional responses to active ZIKA virus.

## Introduction

Zika virus (ZIKV) is a flavivirus primarily transmitted by *Aedes* mosquitoes and sexual contact^1–3^. ZIKV infection during pregnancy has been associated with severe adverse outcomes including microcephaly, intracranial calcification, growth restriction, optical nerve damage, and fetal demise^4,5^. Pathology accompanies the presence of ZIKV in human amniotic fluid, fetal brain, and placenta^6,7^. The mechanisms of ZIKV infection and vertical transmission are complex and appear dependent on multiple variables^8^.

Evidence from animal and cell culture models suggest that ZIKV passes to the fetus by infecting the extraembryonic trophoblast and the placenta later in gestation^9–12^. Experimental models have also revealed several molecular mechanisms related to placental infection and injury^13^. Translating these findings is challenging due to the unique characteristics of the human placenta^14^. Human placenta samples are also generally acquired following delivery, well past the phases of infection and fetal injury. The inaccessibility of human placenta samples during acute phases of infection makes vertical transmission difficult to understand and corroborate with experimental findings. Consequently, postpartum placental samples may not reflect the initial mechanisms of placental cell invasion and replication; elements critical for understanding and preventing vertical transmission.

The heterogeneity of ZIKV associated outcomes confounds the underpinnings of placental infection. Around 1 in 10 pregnancies confirmed positive for ZIKV develop noticeable birth defects^15,16^. The risk and severity of complication decreases with the progression of pregnancy^17^. However, almost half of ZIKV infections are asymptomatic and therefore the extent of afflicted pregnancies remains debated^18^. ZIKV infection can also persist in placentas to term even when no longer detectable in maternal blood and in cases that tested negative^19,20^. Such irregularities in symptoms and outcomes suggest that vertical transmission is not only contingent on the timing of infection but more importantly individual factors. Maternal humoral responses appear to influence adverse pregnancy outcomes related to ZIKV infection^21^. However, the impact of maternal factors on human susceptibility to vertical transmission are not readily distinguishable from intrinsic placental responses.

While maternal humoral factors can influence ZIKV pathogenesis in human placentas, intrinsic responses may explain why a large proportion of infected pregnancies are absent fetal complications. We hypothesize that human placentas respond to ZIKV infection independently of maternal influence and that specific responses might link with pathology. To determine placental-specific responses to viral infection, we examined ZIKV exposure in the absence of maternal factors utilizing a human dual-cotyledon, dual-perfusion model^22,23^. This platform represents a living system with intact maternal-fetal barrier, immune system, and functional vasculature^22,23^. Importantly, two cotyledons from the same human placenta are compared simultaneously. Other key elements of this assay include the removal of maternal blood (and related factors) and the monitoring of placental function during ZIKV exposure.

We compared active and ultraviolet light (UV)-inactivated ZIKV (iZIKV) to gain insight on immune and infection-related responses associated with viral replication. To distinguish specific responses to ZIKV and iZIKV, virus exposed cotyledons were perfused in parallel to unexposed cotyledons from the same placentas. Term placentas delivered by cesarean from uncomplicated pregnancies were used to avoid confounders and labor-related changes^24^. Term placentas are prophetically less susceptible to ZIKV infection as compared to earlier in gestation^25^. We postulated that a lack of infection is indicative of intrinsic mechanism related to placental maturity. Conversely, the presence of ZIKV infection and injury would suggest that human placentas are vulnerable absent maternal humoral factors that may facilitate resistance in vivo.

## Results

### ZIKV exposure invokes subtle physiological and metabolic changes

**Fig. 1:**
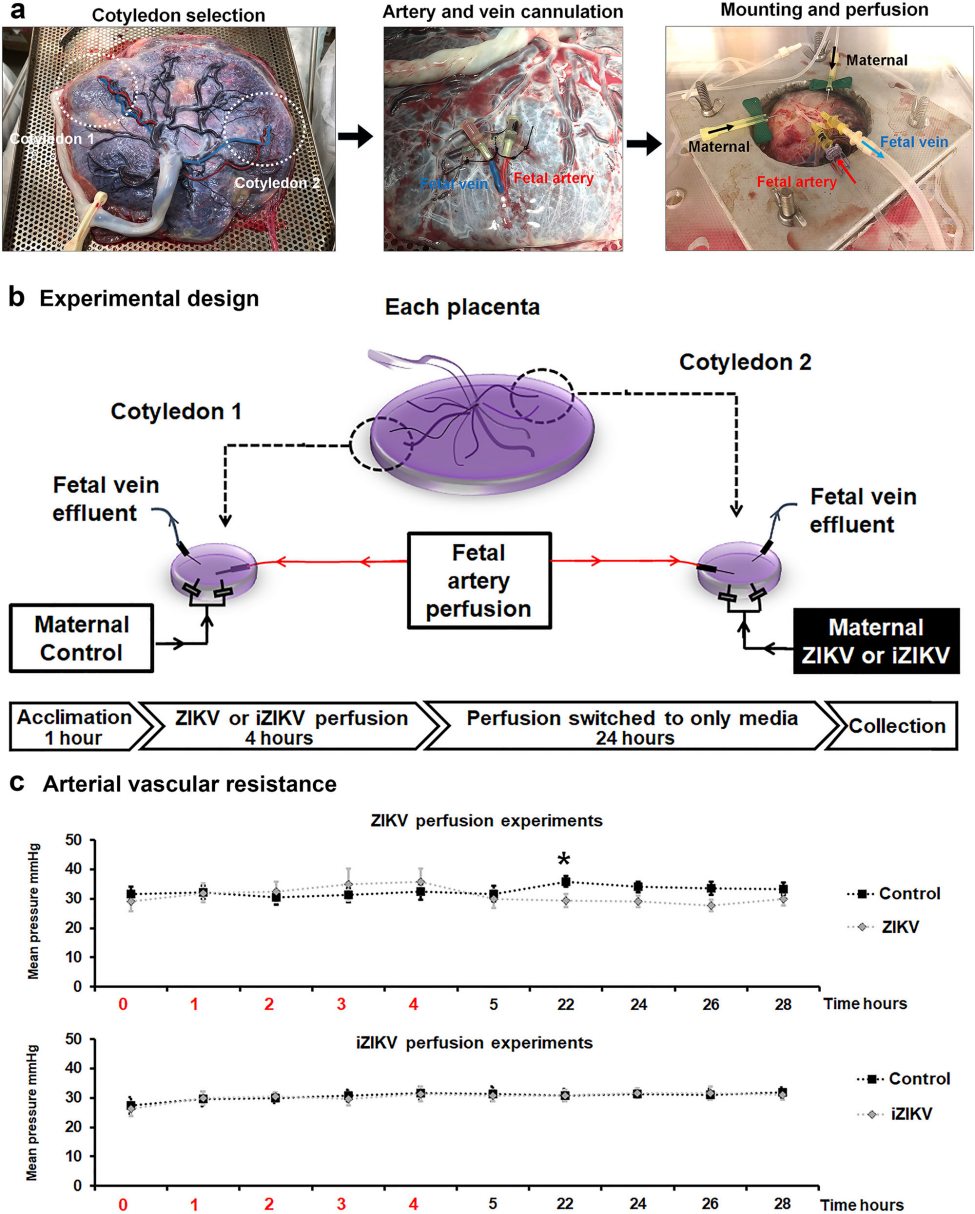
Experimental configuration and design. **a** Representation of cotyledon selection, cannulation, mounting, and the configuration for perfusing fetal vessels and the intervillous space. Arrows indicate direction of flow. Blue denotes the main cotyledon fetal vein, red the fetal artery, and black the intervillous space (maternal side). **b** Experimental design for the dual-cotyledon, dual-perfusion assay. Active ZIKV or UV-inactivated ZIKV (iZIKV) virus was infused into the maternal side of experimental cotyledons. In parallel, control cotyledons isolated from the same placentas are infused with media only. Media perfused into the fetal circuit through arterioles (red arrows), effluxes from fetal veins (blue arrows). Following 4 h of viral exposure, cotyledons were perfused with media only for an additional 24 h before biopsy collection **c** The mean arterial pressures (mmHg) recorded over time for ZIKV (*n* = 10) *vs*. iZIKV (*n* = 9) perfusion experiments. Red numbers along the x-axis indicate the timeframe of viral exposure for ZIKV and iZIKV infused cotyledons. * denotes *p* < 0.05 by unpaired student’s *t*-test and error bars represent the standard error of the mean.

We blindly collected placentas from uncomplicated cesarean delivers for exposure to ZIKV or iZIKV in our dual-cotyledon, dual-perfusion system. Placentas utilized for ZIKV and iZIKV perfusions (Fig. 1a, b) show similar characteristics, with two exceptions (Supplementary Data 1)). Inadvertently, there is a significant difference between maternal race and fetal sex between placentas utilized for ZIKV vs. iZIKV perfusion experiments. The majority of iZIKV-perfused placentas are from deliveries with female offspring, whereas ZIKV-perfused placentas are mostly from Caucasian mothers. Other characteristics, such as Body Mass Index (BMI), gestational age, and birth weight, are not significantly different between groups.

Physiological monitoring of fetal artery pressures show perfusion with ZIKV slightly but significantly reduces vascular tone at 22 h as compared to control-perfused cotyledons from the same placentas (Fig. 1c). Changes in arterial pressure are not observed in cotyledons perfused with iZIKV relative to the control-perfused cotyledons. Metabolic analysis of the venous effluents collected at the end of each experiment show no difference between control and ZIKV-perfused cotyledons (Supplementary Information). Importantly, lactate values being low and similar among groups indicates that cotyledons were metabolically viable^26^. The only observed change is the partial pressure of oxygen (PO_2_) for virus exposed and control cotyledons as compared to PO_2_ levels of the media prior to arterial perfusion. This may relate to oxygen consumption and dilution as the buffer passes through the cotyledons vasculature.

### ZIKV infection in perfused human cotyledons

**Fig. 2:**
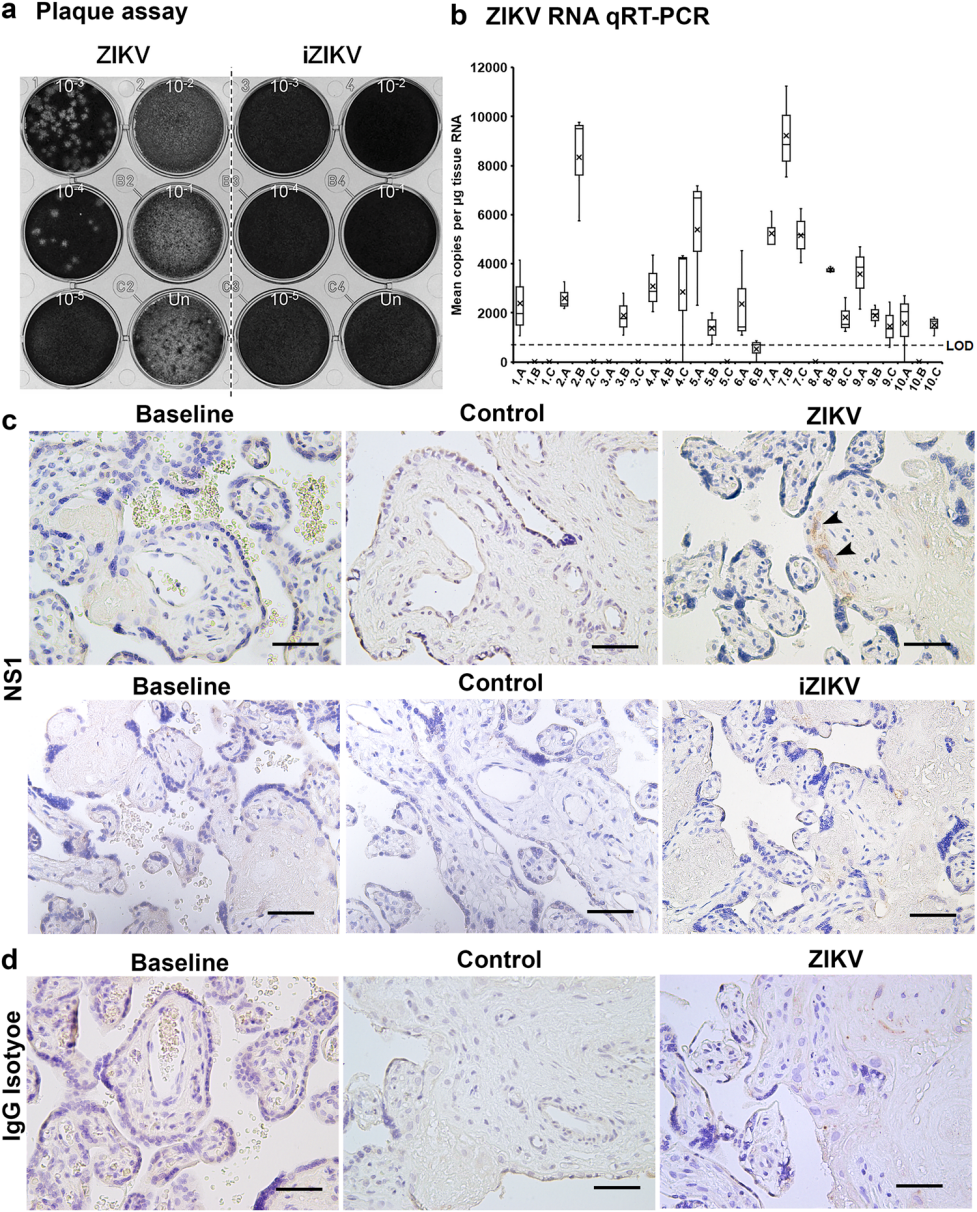
ZIKV infection in perfused cotyledons. **a** Plaque assay validation of viral stocks depicted in grayscale. Vero cells were inoculated with 100 µl of Undiluted (Un) and as annotated 10-fold serial dilutions of ZIKV and iZIKV in parallel. **b** Quantitative real-time PCR (qRT-PCR) analysis for ZIKV RNA. A-C represent the three biopsies collected and analyzed from each ZIKV perfused cotyledon (1-10). Box-plots represent the median in the centerline, upper and lower quartiles at top and bottom of box, min/max points as whiskers and the mean as x. LOD represents the limit of detection. **c** Staining for ZIKV NS1 in samples collected at baseline and following perfusion. NS1 staining is visualized with DAB in brown (arrowheads). **d** Staining’s conducted in parallel using an IgG isotype in place of NS1. Hematoxylin was used to counterstain nuclei in blue. Scale bars = 50 µm.

**Fig. 3:**
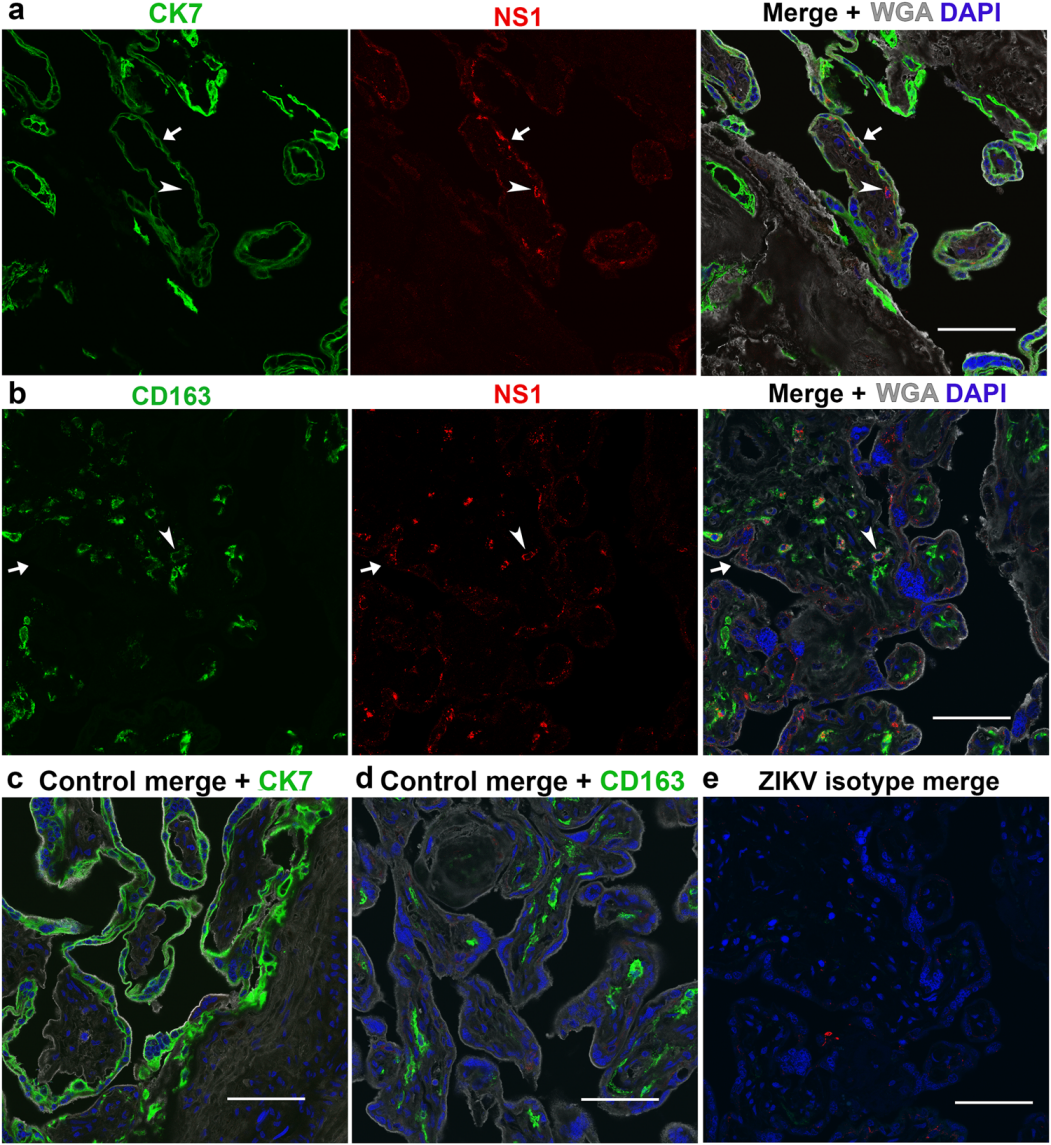
NS1 colocalization in ZIKV perfused cotyledons. **a**–**b** Confocal images acquired from cotyledons exposed to ZIKV. Sections were stained with Cytokeratin 7 (CK7) or CD163 depicted in green, along with NS1 in red, the lectin WGA in gray and DAPI as blue. Arrows denote colocalization of NS1 with CK7 or CD163 positive cells, respectively. Arrowheads denote NS1 staining that does not colocalize. **c**–**d** Merged images of NS1, WGA, DAPI, and CK7 or CD163 staining’s in control cotyledons perfused in parallel to ZIKV. **e** Representative image of serial sections from ZIKV exposed sample stained with IgG isotype antibodies in place of anti-CK7, CD163 and NS1. Scale bars = 50 µm.

**Fig. 4:**
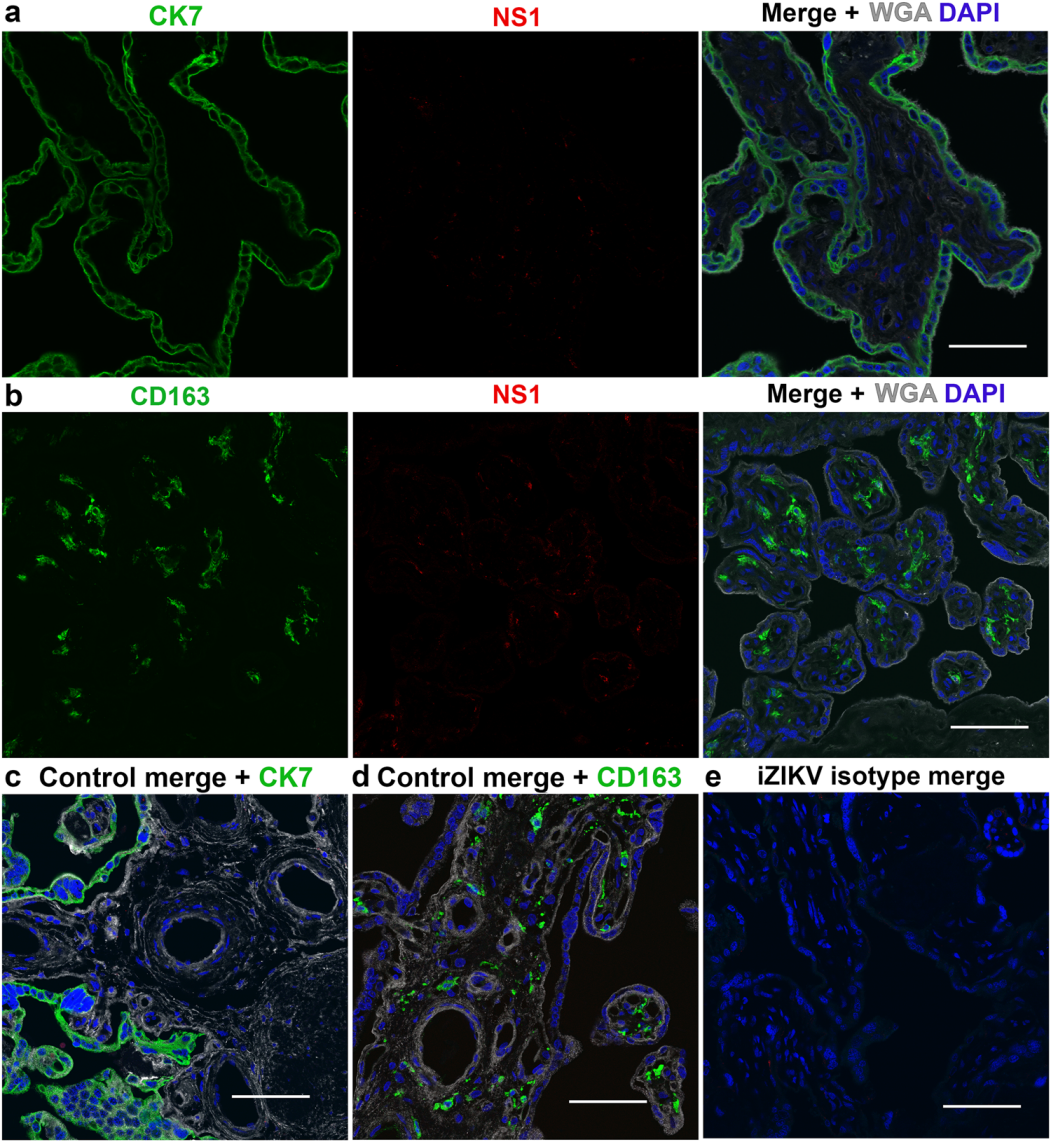
Absence of NS1 in iZIKV perfused cotyledons. **a**–**b** Confocal images acquired from cotyledons exposed to iZIKV. Parallel to Fig. 4 sections were stained with Cytokeratin 7 (CK7) or CD163 depicted in green, along with NS1 in red, the lectin WGA in gray and DAPI as blue. **c**–**d** Merged images of NS1, WGA, DAPI, and CK7 or CD163 staining’s in control cotyledons perfused in parallel to iZIKV. **e** Representative image of serial sections from iZIKV exposed samples stained with IgG isotype antibodies in place of anti-CK7, CD163 and NS1. Scale bars = 50 µm.

Taking into account that iZIKV lacked infection potential by plaque assay (Fig. 2a), the presence of viral RNA was only measured in ZIKV exposed samples. Postperfusion quantitative real-time PCR (qRT-PCR) analysis of three biopsies per ZIKV exposed cotyledon reveals the presence of viral RNA (Fig. 2b). The viral RNA copy numbers are highly variable and not significantly different (*p* > 0.05 by one-way ANOVA) between biopsies or cotyledons. Immunohistochemistry for NS1 corroborates the presence of ZIKV in exposed cotyledons (Fig. 2c). NS1 was absent in both control and baseline samples, suggesting the antibody is specific (Fig. 2d). The IgG isotype control validates the lack of non-specific binding in these samples (Fig. 2c). Confocal microscopy reveals colocalization of ZIKV NS1 with Cytokeratin 7 and CD163, markers of placental trophoblasts and Hofbauer cells, respectively (Fig. 3a, b)^27,28^. NS1+/Cytokeratin 7+ and NS1+/CD163− cells were restricted to the syncytium whereas all NS1+/CD163+ cells were within the villous stroma. Once more sections from control-perfused samples (Fig. 3c, d) and ZIKV exposed samples given the IgG isotypes in lieu of the primary antibodies (Fig. 3e) lacked positive staining. Analogous to these results no positive staining is observed in iZIKV perfused samples and their respective controls (Fig. 4).

### Syncytium injury and cell death result from ZIKV exposure

**Fig. 5:**
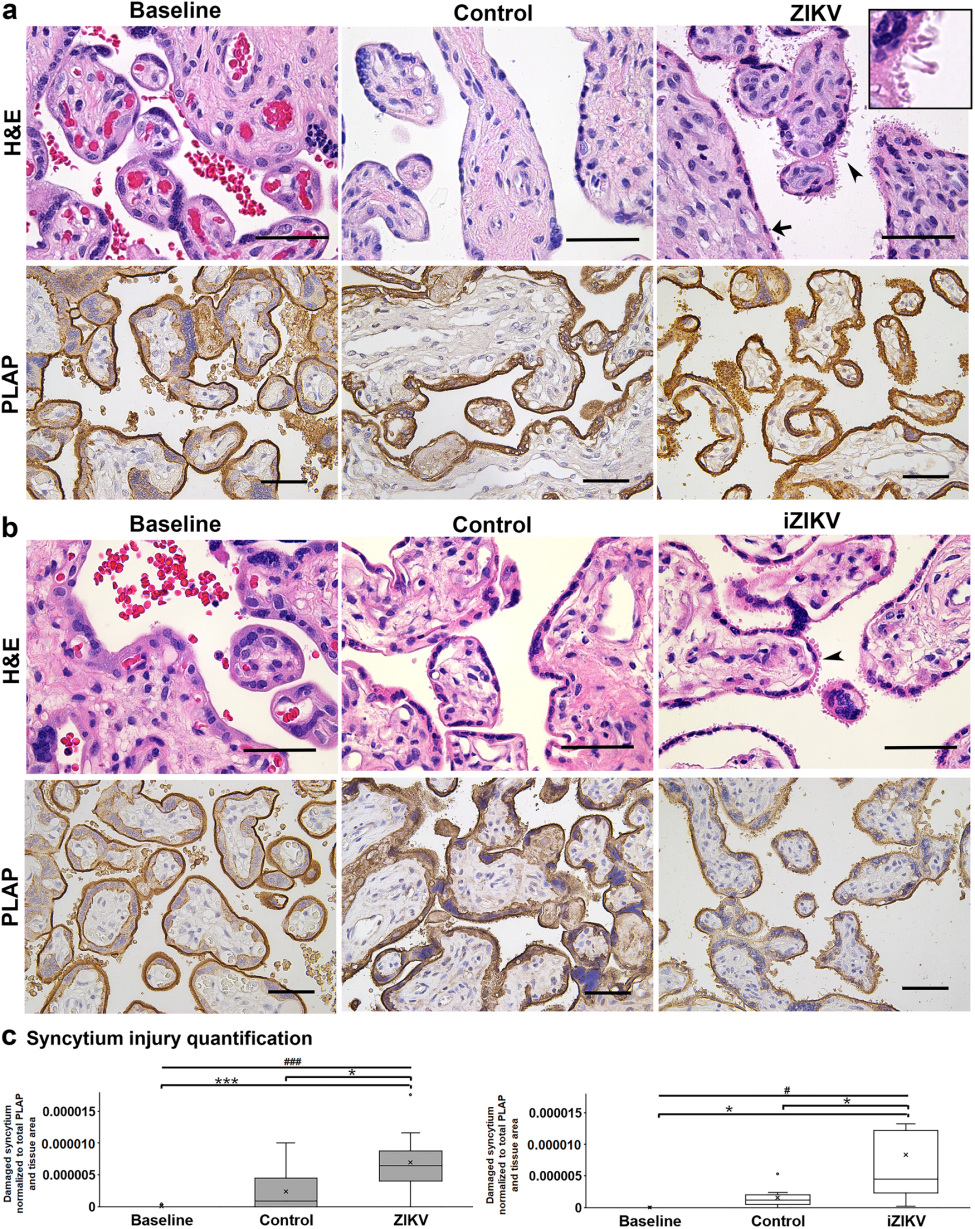
ZIKV and iZIKV invoke syncytium injury. H&E and PLAP staining representing syncytium remodeling (arrowheads) at Baseline and following exposure to ZIKV (**a**) and iZIKV (**b**). In the ZIKV panel, an arrow denotes normal syncytium and the inset (right corner) magnifies the damaged region designated by the arrowhead. PLAP staining is represented by DAB in brown. Scale bars = 50 µm. **c** Quantification of syncytium remodeling in PLAP stained sections normalized to the total area of tissue within each image analyzed. The left graph represents *n* = 10 and right graph *n* = 9 samples per condition. Box-plots represent the median in the center line, upper and lower quartiles at top and bottom of box, min/max points as whiskers, outliers as dot, and the mean with x. ^#^*p* < 0.005, ^###^*p* < 0.0005 by one-way ANOVA. *p < 0.05, ***p < 0.0005 by Tukey’s post-hoc test.

Histopathology analysis of perfused tissue reveals damage to the placental barrier with ZIKV (Fig. 5a) and iZIKV (Fig. 5b). The injury represents PLAP+ membrane particles (aka exosomes or blebs) shedding into the intervillous space from the syncitium^29^. The presence of syncytium injury is significantly greater with ZIKV and iZIKV exposure compared to controls and aseline samples (Fig. 5c), suggesting that cellular damage occurs irrespective of the virus status.

**Fig. 6:**
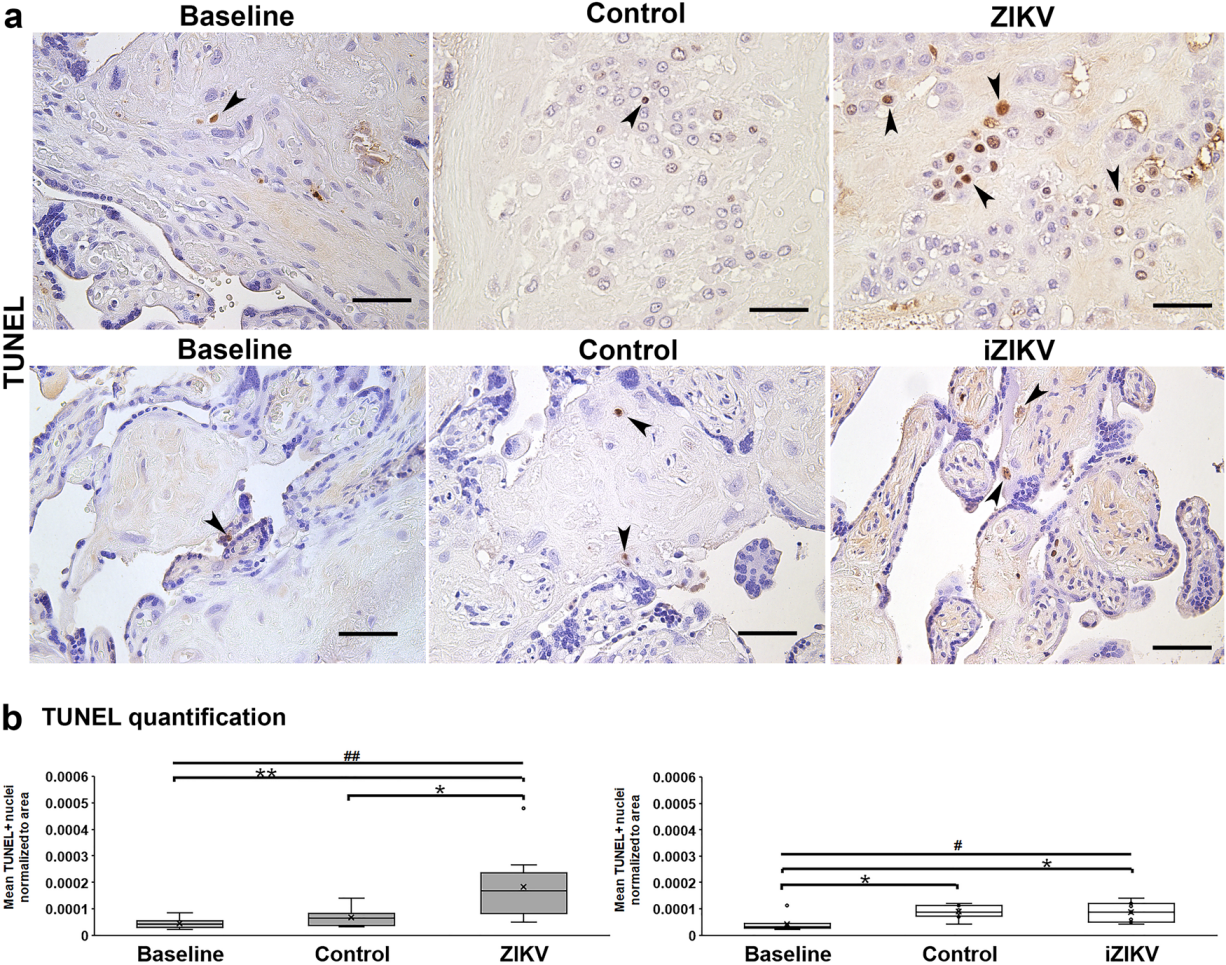
Cell death elevated with ZIKV perfusion. **a** Representative TUNEL staining with DAB in Baseline, Control, ZIKV, and iZIKV exposed samples. TUNEL positive cells (arrowheads) appear as brown nuclei *vs*. blue nuclei counterstained with hematoxylin. Scale bars = 50 µm. **b** Quantification of TUNEL positive nuclei normalized to the area of tissue within each field analyzed. The left graph represents *n* = 10 and right graph *n* = 9 samples per condition. Box-plots represent the median in the centerline, upper and lower quartiles at top and bottom of box, min/max points as whiskers, outliers as dot, and the mean with x. ^#^*p* < 0.005, ^##^*p* < 0.005 by one-way ANOVA. *p < 0.05, **p < 0.005 by Tukey’s post hoc test.

**Fig. 7:**
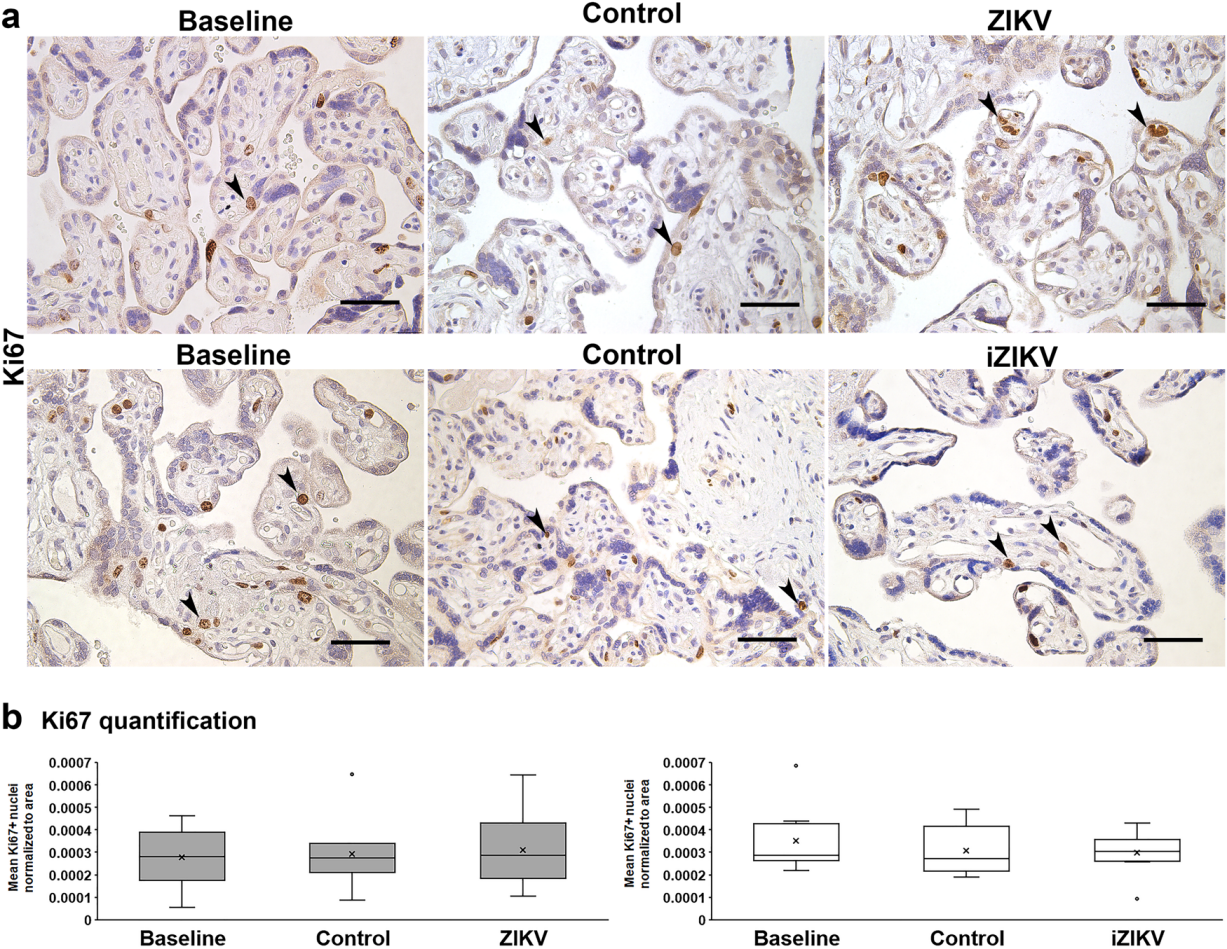
Cell division unaltered in perfused cotyledons. **a** Representative of Ki67 staining with DAB and hematoxylin in Baseline, Control, and virus exposed samples. Scale bars = 50 µm. Ki67 positive cells (arrowheads) appear as brown nuclei *vs*. blue nuclei counterstained with hematoxylin. **b** Quantification of Ki67 positive nuclei normalized to the area of tissue within each field analyzed. The left graph represents *n* = 10 and right graph *n* = 9 samples per condition. Box-plots represent the median in the centerline, upper and lower quartiles at top and bottom of box, min/max points as whiskers, outliers as dot, and the mean with x.

**Fig. 8:**
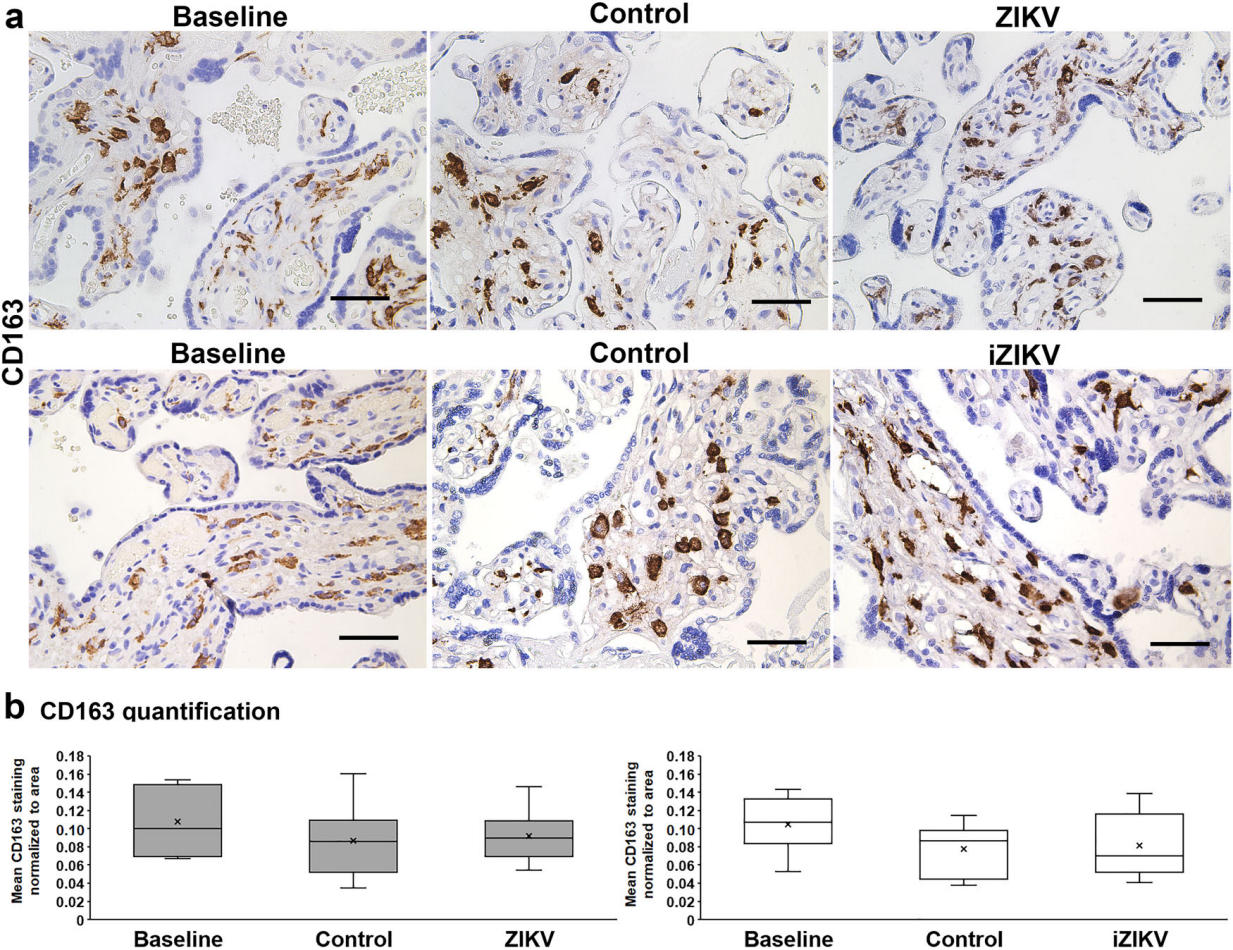
No change in Hofbauer cells with perfusion. **a** Representative CD163 staining with DAB and hematoxylin in Baseline, Control, and virus exposed samples. Scale bars = 50 µm. **b** Quantification of CD163 staining normalized to the area of tissue within each field analyzed. The left graph represents *n* = 10 and right graph *n* = 9 samples per condition. Box-plots represent the median in the centerline, upper and lower quartiles at top and bottom of box, min/max points as whiskers, outliers as dot, and the mean with x.

To evaluate if placental barrier injury corresponds with changes in key cellular processes, we quantified indicators of cell death (TUNEL), cell division (Ki67), and cellular-mediated inflammation (CD163) from all perfusion experiments. CD163 is present on Hofbauer cells and released in soluble form with inflammation^30^. Thus CD163 staining may reflect placental Hofbauer cell numbers and their status. With ZIKV, more regions containing TUNEL+ nuclei are visible (Fig. 6a)^31,32^. A significant increase of TUNEL+ cells occurs with ZIKV exposure (Fig. 6b). Samples exposed to iZIKV are not significantly different versus controls, but there is a difference in comparison to baseline samples. There is no such pattern with Ki67+ nuclei marking proliferating cells (Fig. 7) or CD163+ cells (Fig. 8) between no differences are observed with Ki67 or CD163 staining’s between perfusion conditions (Figs. 7b and 8b).

### ZIKV infection and pathology correspond with unique transcriptional changes

**Fig. 9:**
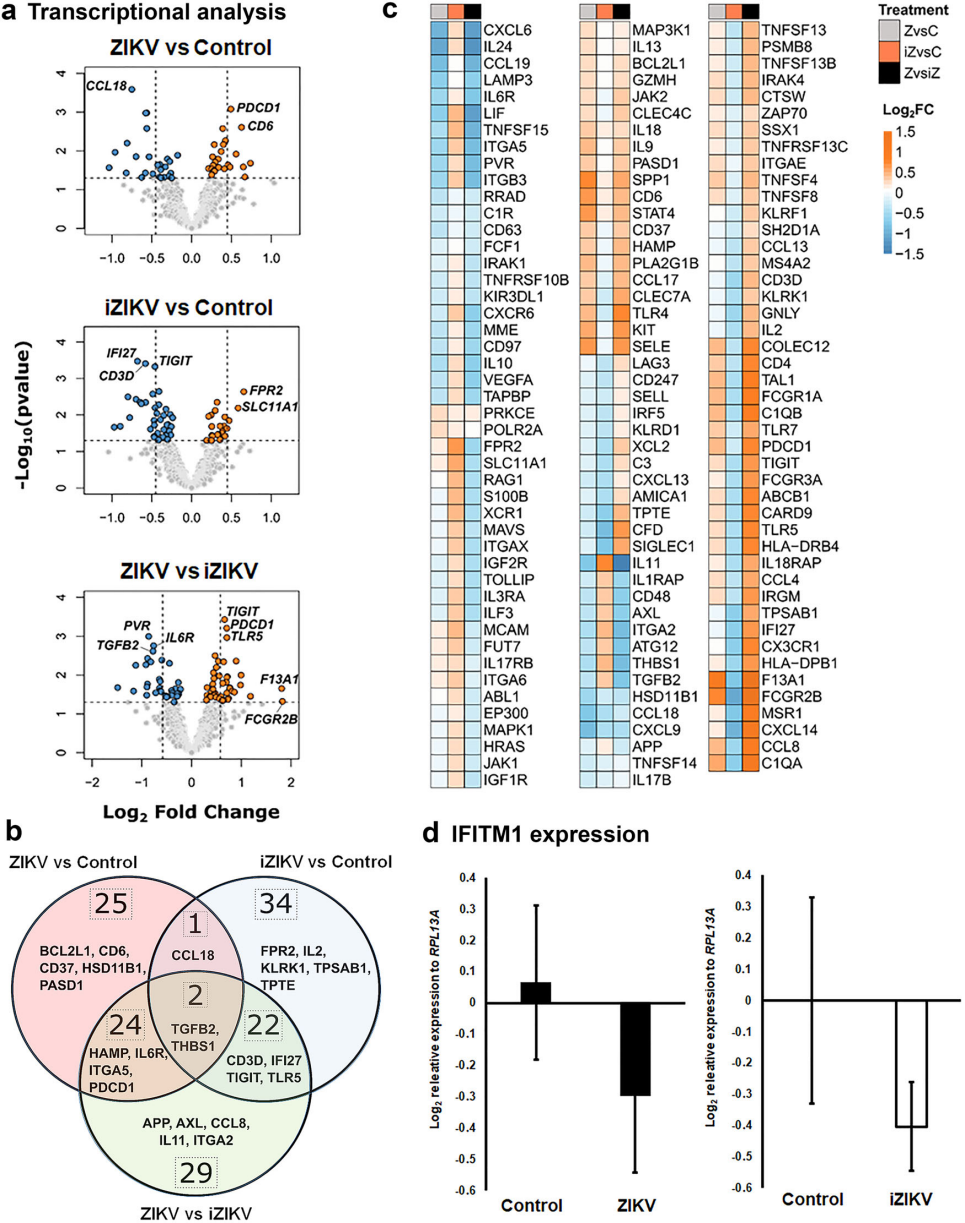
Transcriptional changes with ZIKV and iZIKV perfusion. **a**–**c** nCounter transcriptional analysis in virus perfused *vs.* Control cotyledons (*n* = 5 samples per condition). **a** Volcano plots of differentially expressed (DE) genes among respective comparisons. Points on the plots represent individual genes. Hashed lines denote a *p* value < 0.05, absolute log_2_ fold change > 0.45 for ZIKV and iZIKV vs. Controls, and absolute log_2_ fold change > 0.58 for ZIKV *vs*. iZIKV wherein the top genes are annotated. Differentially expressed (DE) genes significantly upregulated are shown in red while those downregulated are blue. (**b**) Venn diagram annotating the total number and overlap for significant DE genes between comparisons. The top five most significant genes based on the limma moderated *t*-statistic are annotated. **c** Heatmaps denoting all significant (*p* < 0.05) DE genes, clustered by complete linkage on Euclidean distances of log2 fold change values. **d** The relative expression of *IFITM1* by qRT-PCR in most of the same samples (*n* = 5–7 per condition) examined by the nCounter. Control samples reflect cotyledons from the same placentas used for either ZIKV or iZIKV perfusions respectively. Box-plots represent the median in the centerline, upper and lower quartiles at top and bottom of box, min/max points as whiskers, and the mean with x.

To understand the mechanisms of ZIKV infection and pathology, we used the NanoString nCounter gene expression assay to investigate immune-related and adhesion molecule gene signatures. The NanoString Human PanCancer Immunology Panel has been used for the immunologic characterization of peripheral blood of ZIKV-infected patients^33^. Differential expression (DE) analysis of each virus perfusion was compared to respective control cotyledons. We also investigated DE genes by direct comparison of ZIKV and iZIKV perfusions^33^. With few exceptions, the most prominent DE expressed genes do not overlap between ZIKV vs. control as compared to iZIKV vs. control (Fig. 9a and b). Mainly chemokine *C-C Motif Chemokine Ligand 18 (CCL18)*, *Transforming Growth Factor Beta 2 (TGFB2)* and *Thrombospondin 1 (THBS1)* were related to ZIKV and iZIKV when compared to their respective controls (Fig. 9b). The pattern of expression (upregulation vs. downregulation) contrasts between samples infected with ZIKV as compared to those exposed to iZIKV (Fig. 9b and c). Among these genes, *AXL Receptor Tyrosine Kinase* (*AXL)* and *Toll-Like Receptors* (*TLRs)* have been implicated for ZIKV infection^34–36^. With ZIKV infection, several genes related to innate immunity and Hofbauer cells are reduced or downregulated in expression compared to controls. Notably *C-X-C Motif Chemokine Ligand 6* (*CXCL6), Interleukin 6 Receptor (IL6R)*, and *Interleukin 24* (*IL24)* are lower with ZIKV infection^37–39^. Genes increased or upregulated in expression include key regulators of immune responses, *PDCD1 and TLR5*^40,41^. A complete list of significant DE genes is provided in the Supplementary Data 2 file.

**Fig. 10:**
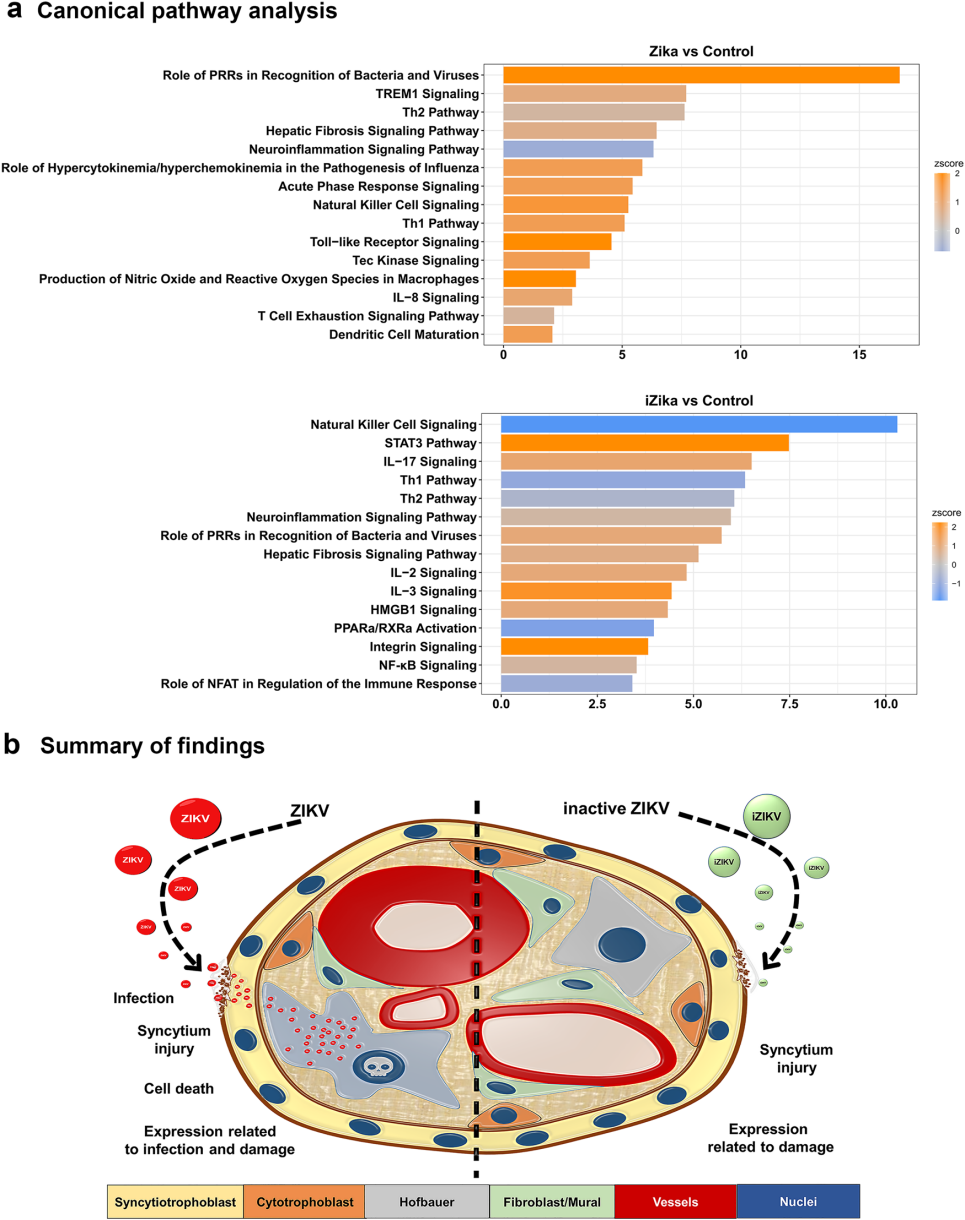
Network analysis and synopsis. **a** Bar charts of significantly enriched IPA canonical pathways for ZIKV vs. control (top graph) and iZIKV vs. control (bottom graph) DE genesets. Bars represent each canonical pathways *p* value on a negative logarithmic scale. The top 15 pathways related to immunity and inflammation are ranked based on *p* value ≤ 0.05. Bars are shaded according to their z-score activity predictions. Orange represents pathways with positive z-scores and depicts pathway activation; blue represents pathways with negative z-scores and depicts pathway inactivation. PRRs denote Pattern Recognition Receptors. **b** Summary of findings and potential model of ZIKV transmigration across the maternal-fetal interface.

In spite of these differences, we did not observe changes in interferon-stimulating genes (ISG) as anticipated with ZIKV exposure^42^. Therefore, we examined the expression for an indicator of interferon responses, *Interferon Induced Transmembrane Protein 1* (*IFITM1)* by qRT-PCR. The expression of *IFITM1* was lower between controls and ZIKV or iZIKV exposed samples but this was not significant (Fig. 9d). Being that interferons do not appear robustly activated, we conducted canonical pathway analysis using IPA to understand the immune and inflammatory processes related to the observed transcriptional patterns (Fig. 9). To distinguish differences between infection and exposure, this analysis was conducted for ZIKV and iZIKV vs. their respective controls. With ZIKV, most of the top-ranking pathways are activated and include the Role of Pattern Recognition Receptors in Recognition of Bacteria and Viruses, Triggering Receptor Expressed on Myeloid cells 1 (TREM1) Signaling, and the T helper type-2 (Th2) Pathway (Fig. 10a). A greater number of pathways are inactivated with iZIKV, notably Natural Killer Cell Signaling, which is activated by ZIKV (Fig. 10a). Th1 and Th2 pathways were also inactived with iZIKV but active with ZIKV. In contrast, the Neuroinflammation Signaling Pathway was inactive with ZIKV but active with iZIKV. Only the Role of Pattern Recognition Receptors in Recognition of Bacteria and Viruses and Hepatic Fibrosis Signaling Pathways were similar in activation status between ZIKV and iZIKV. The remaining relationships were group specific. Notable inflammatory pathways activated with ZIKV include Toll-like Receptor, IL-8 Signaling and Production of Nitric Oxide and Reactive Oxygen Species in Macrophages. In contrast, STAT3, IL-2, IL3, and IL-17 Signaling were activated with iZIKV.

## Discussion

Maternal symptoms related to ZIKV infection are heterogeneous or often absent^43^. There is no association between maternal health status and fetal complications related to ZIKV^44^. However, inflammatory factors are elevated in maternal blood in pregnancies complicated by ZIKV^45^. Humoral factors may not associate with human maternal health, but they appear to influence adverse pregnancy outcomes associated with congenital ZIKV syndrome (CZS)^21^. Evidence from animal models suggests this to be the case^46^. Yet, the influence of maternal factors on human placental immunity is not easily distinguishable from intrinsic responses.

Our study demonstrates that ZIKV can infect human term placentas in the absence of maternal factors. Signs of infection and pathology were present in all cotyledons exposed to the active ZIKV. These results suggest that maternal humoral factors may be protective, particularly since fetuses in the third trimester and by extension their placentas, are believed to be less susceptible^17^. The ability of ZIKV to infect and damage perfused term human cotyledons, in an experimental model free of maternal influence supports this possibility. This notion coincides with the observation that the majority of ZIKV infected pregnancies are absent complications. In our experiments, ZIKV exposed cotyledons were afflicted in a virus-specific manner. However, the threshold for placenta injury, including cell death and syncytium injury, in relation to ZIKV transmission remains unknown.

Crosstalk between maternal and fetal responses to ZIKV may govern placenta immunity and vertical transmission. Irregularities of ZIKV related outcomes in dizygotic twins suggest that individual fetal components are involved^47^. The placenta is fetal-derived tissue and may react differently to ZIKV and maternal factors depending on genetic and epigenetic factors. Though we did observe infection and injury in all placentas exposed to ZIKV, the observed variability may relate to individual genetic or clinical characteristics. Therefore, certain fetal and maternal predispositions may coalesce to enable ZIKV transmission.

During pregnancy the placenta develops predominately innate immune components whereas adaptive immunity is mainly maternal^12^. Fetal-maternal symbiosis involves declines in adaptive immune cells and function^48^. This process enables fetal tolerance at the cost of increased maternal vulnerability to infection^49^. Incidentally, maternal humoral factors are important component of fetal protection that can also lead to complications. Specifically, immunoglobulin G (IgG) antibodies cross the placenta early in pregnancy to provide passive immunity but can also invoke fetal autoimmune disorders^50,51^. Within the context of ZIKV, evidence suggests that transplacental IgGs enhance fetal infection and promote adverse outcomes^21^. However, this may depend on the timing of infection; being permissive earlier in gestation and perhaps protective as the placenta and its innate immune system matures.

In conjunction with maternal humoral factors, the fetus relies on the placental barrier and its innate immune defenses for preventing vertical transmission of pathogens. Studies have implicated placental innate immune regulation as a factor in placental susceptibility to ZIKV infection^52–54^. Our experimental evidence suggests that within intact cotyledons, human placenta cells are susceptible to ZIKV (Figs. 2 and 3). This coincides with cell death and the downregulation of specific innate immune genes that may alter placental function and viral resistance (Figs. 6 and 9)^10^. In contrast, syncytiotrophoblast injury occurred with both active and inactive ZIKV exposure (Fig. 5), suggesting that viral products can elicit placenta barrier compromise, independent of infection^55^. This event may enable access to intervillous cells including Hofbauer cells that are vulnerable to ZIKV infection^25,53,56^.

Hallmarks of ZIKV infection associated with Hofbauer cell infection are signatures of innate immune and antiviral responses^57^. Innate immune activation takes place following ZIKV infection. This process includes the acute virus-induced expression of a variety of antiviral and immune-modulatory genes. Activation is succeeded by the production of types I and III interferons and their induced expression of ISGs^58^. While our gene expression analyses identified innate immune and inflammatory gene regulation following brief exposure or early infection by ZIKV, we did not identify robust ISG activation in our model. Variations in experimental design and our use of perfused tissue vs. cultured cells may account for this outcome. Additionally, the aforementioned studies observed ISG expression beyond 24 h of ZIKV exposure. It is likely that the differential expression of the immune and inflammatory genes shown here precedes ISG activation in the context of placental ZIKV exposure or infection. This coincides with the lack of cell division or increase of Hofbauer cells (Figs. 7–8) that are observed in vivo and may relate to ISG activation and/or maternal stimuli^59^.

In our comparison, it is possible that the few mutually expressed genes between active and inactive ZIKV exposure support syncytium injury. Alternatively, syncytium injury can occur by different mechanisms initiated by either infection or host interactions. Tissue damage is observed in ZIKV infected placentas and experimental animal models have linked this pathology to type I interferon signaling^60–62^. Pregnancy complications such as growth restriction arise with ZIKV infection and accompany placental damage^63,64^. Syncytium injury also manifests in pregnancies complicated by preeclampsia or growth restriction with no known infections^65^. In placentas with ZIKV infection or unrelated pregnancy complications, syncytium injury is accompanied by the activation of inflammatory pathways such as TLR and TNF^66–68^. Therefore, syncytium injury may be a consequence of inflammation provoked by pathogen exposure or disease. Syncytiotrophoblast molecular pattern sensing likely initiate such destructive inflammatory responses to combat infection and signal localized and systemic (maternal) defenses. This process may precede the stimulation of placental ISGs, which perhaps activate via a combination of endogenous and circulating factors. Our observations support this possibility as pathogen-related pattern recognition and the activation of several inflammatory pathways accompanied ex vivo syncytium injury (Figs. 5 and 10a).

Taken that not all placental cells are infected yet may react to virus exposure, the responses to ZIKV exposure and infection may not be mutually exclusive. The downregulation of certain genes such as *Interferon Alpha Inducible Protein 27* (*IFI27)* with virus exposure supports this possibility as it is upregulated in ZIKV infected human cell cultures^69^. Another possibility is that genes altered by virus exposure are suppressed during ZIKV infection through viral-directed processes^42^. The downregulation of inflammatory genes *CCL18*, *CCL19*, *CXCL6*, *CXCL9*, *IL6R*, *IL11*, *IL24*, and *Integrin Subunit Beta 3* (*ITGB3)* with ZIKV infection may reflect placental immune tolerance. (Fig. 9c, Supplementary Data 2). ZIKV infection also resulted in the upregulation of *Programmed cell death protein 1* (*PDCD1*), a gene implicated in immune suppression and cell death^40^. This trend supports the ability of ZIKV to alter immunity and attenuate innate immune programming to facilitate viral replication and spread^52,57,70^.

Notably, we detected the downregulation of two genes, *Leukemia Inhibitory Factor* (*LIF*) and *Poliovirus Receptor* (*PVR)*, related to other viruses yet not currently linked to ZIKV infection. In human placentas, LIF is associated with HIV inhibition^71^. In contrast, *PVR* (aka *CD155)*) the canonical poliovirus receptor, is expressed with no known role in the human placenta^72,73^. Additional investigation and validation are necessary for understanding these patterns of gene expression and conserved mechanisms related to viral infection and pathology in human placentas.

Our transcriptional analysis reveals distinct patterns of expression between ZIKV infection and exposure. Canonical pathways related to the transcription profiles showed little overlap between ZIKV and iZIKV. However, in both cases these pathways reflect innate immunity and inflammatory responses (Fig. 10a). Pathways activated by ZIKV and iZIKV include pattern recognition, which may mediate innate defenses that trigger inflammation and cellular injury. Interestingly, Natural Killer (NK) Cell Signaling was active with ZIKV but inactive with iZIKA. This suggests the virus can be immunosuppressive and that the process of infection provokes inflammatory cell death^74^. With infection, placental cells may also be eliciteing maternal reinforcements in the form of NK cells, which are elevated with ZIKV viremia^75^. However, further investigation is warranted to distinguish if such mechanisms combat transmission by signaling maternal inflammation and eliminating infected cells or if they create a permission microenvironment for ZIKV to reach the fetal bloodstream.

Regarding the investigation of ZIKV infection in human placentas, our study has several strengths. Foremost is the use of the human dual-cotyledon, dual-perfusion assay. For examining placental viral infection, this platform offers several advantages: 1. In every perfusion experiment two functional cotyledons from the same placenta are run in parallel (control and virus exposed), 2. Simulating in vivo conditions the vasculature and intervillous space are perfused simultaneously, and 3. The absence of circulating humoral factors permits the examination of maternal influence (or rather lack of) on placental infection and injury. Our system is also aseptic, viable, and validated. We optimized our perfusion system to run for over 28 h and confirmed the presence of ZIKV infection by qRT-PCR and immunostaining’s. The absence of vasospasm and metabolic changes, specifically in lactate, substantiate the viability of perfused cotyledons^26^. Finally, the comparison of ZIKV vs. iZIKV enables the distinction of responses related to infection and immunity.

Our study is not without limitations. The first is the predominance of placentas from Caucasian donors with ZIKV perfusions and the fetal sex being mostly female with iZIKV perfusions. This was unintended as experiments are initiated without knowledge of race or fetal sex. In turn, clinicians that consented donors and collected placentas were unaware of the exact virus perfusion treatment. Finally, we received clinical characteristics only when perfusion experiments were successful with placentas free of leakage or damage from the cesarean or processing. We acknowledge differences in clinical characteristics may bias comparisons between active and inactive ZIKV perfusions. However, most comparisons are between cotyledons from the same placentas and likely uninfluenced by such potential confounders.

The main limitation of our study is the use of the term placentas that may not represent events at earlier stages of pregnancy. To date several studies have demonstrated an association between complications and gestational age, providing a link between developmental maturity and infection^17,76^. Not all studies correlate adverse outcomes with infection earlier in gestation^64,77^. Furthermore, the majority of ZIKV infected pregnancies even in the first trimester appear unaffected. Paradoxically, ZIKV infection has been identified in placentas of uncomplicated pregnancies^25^. Such cases lack placental pathology. In contrast, ZIKV infected pregnancies with adverse fetal outcomes exhibit placental pathology including syncytium injury^78^. Therefore, placental injury is perhaps a determinant of vertical transmission.

The symbiosis between two genetically distinct immune systems (maternal and fetal) is a unique biological process that occurs naturally only in pregnancy. Our understanding of vertical transmission is complicated by the immunological environment of pregnancy; a delicate balance between fetal protection and tolerance. Maternal-fetal interactions may relate to the ability of ZIKV to compromise the placental barrier in some but not all pregnancies. Based on our results we postulate that this may occur via damage to the placental microenvironment when maternal (protective) factors are absent a. In summary, our study further supports the importance of maternal interactions in combating placental infection and viral transmission to the fetus.

## Methods

### Perfusion experiments

Placenta collections and perfusions were carried out with approval from the Madigan Institutional Review Board. Unlabored placentas were acquired following consent and delivery by cesarean section. Only uncomplicated term pregnancies were included in this study. Pregnancies with complications or with known maternal use of tobacco, drugs or alcohol were excluded. Placentas were processed within 30–60 min of delivery, as previously described^22,23^. Two cotyledons were excised from individual placentas and the main fetal artery and vein were cannulated for perfusion. Two butterfly needles were inserted just below the chorionic plate to simulate maternal perfusion (Fig. 1a). Cotyledons were placed in customized enclosures within an incubator at 36 °C. Cotyledons were continuously perfused with warmed and oxygenated media that was not recirculated, within a laminar hood following BSL2 standards. The media consisted of Hanks’ Balanced Salt solution pH 7.4 (Sigma-Aldrich, St. Louis, MO, USA), containing 2 g/L Albumin (Sigma), 2000 U/L Heparin (Patterson Veterinary Supply, Devens, MA, USA) 5 mg/L Gentamycin, and 0.35 g/L Sodium Bicarbonate (Sigma). The remaining placental tissue was utilized to provide the baseline references. These samples designated as “baseline”, constitute fresh unperfused intervillous biopsies collected as cotyledons designated for perfusion were being prepared.

For the initial acclimation and blood clearance, peristatic pump rates were 4 mL/minute and 10 mL/minute for fetal and maternal sides, respectively, within the first hour of perfusion^22,23^. To make volumes manageable for long-term perfusion, rates were adjusted to 1.6 mL/minute (fetal) and 3.3 mL/minute (maternal) for the remainder of each experiment. Viral exposure was initiated after all blood was visibly cleared, indicated by the blanching of cotyledons and clear effluent leaving the venous lines. Cotyledons were randomized to receive via the intervillous space, the vehicle control, or either ZIKV or iZIKV (Fig. 1b). The iZIKV was derived from the same ZIKV stock (strain Brazil Fortaleza 2015^58^) by exposing the viral stock to ultraviolet (UV) light using a UV-crosslinker fora 30 min. Both ZIKV and iZIKV preparations were validated by plaque assay to confirm infectious titer and lack thereof, respectively. Vehicle control cotyledons referred to as “controls”, were perfused in parallel to experimental cotyledons with the same buffer that lacked the addition of either ZIKV or iZIKV. ZIKV or iZIKV was infused into the intervillous space of experimental cotyledons at a dose of 200 plaque-forming units per mL for 4 h then switched to media for an additional 24 h (Fig. 1c). Clinical characteristics for each placenta/donor were received at the conclusion of each successful perfusion experiments (Supplementary Data 1).

Arterial resistance was monitored by inline transducers to validate perfusions and cotyledon viability during the course of each experiment. At the end of each experiment, venous perfusates and baseline media (unperfused buffer), were collected for metabolic analysis (Supplementary Table 1) with the i-STAT CG4+ system/cartridges (Abbott Laboratories, Lake County, IL, USA). Note, CG4+ measurements for TCO_2_, sO_2_, and BE in our perfusion media were either not detectable or irrelevant due to the removal of erythrocytes and continuous oxygenation of the media. Cotyledons were then biopsied for histological and molecular analyses.

### Immunohistochemistry (IHC)

1 cm x 1 cm biopsies were placed in five times their volume of NBF (Thermo Fisher Scientific, Waltham, MA, USA). Samples fixed at 4 °C for a minimum of 7 days before processing and paraffin embedding. Sections were cut 4 µm thick, cleared and antigen retrieval for most targets was conducted at 110 °C for 15 min using the Decloaking Chamber NxGen (Biocare Medical, Pacheco, CA, USA). Only Ki67 staining’s were conducted using a water bath heated to 95 °C for 15 min. All antigen retrievals were conducted with citrate buffer pH 6.0 (Vector labs, Burlingame, CA, USA) + 0.5% Tween 20 (Sigma). Blocking was conducted with the peroxide blocking kit (Dako-Agilent, Santa Clara, CA, USA) and then with Streptavidin-Biotin kit (Vector) according to manufacturer instructions, before sections were left overnight at 4 °C in PBS + 1% BSA containing 10% Donkey serum (Jackson Immuno Research, West Grove, PA, USA). The next day primary antibodies or isotype controls were applied in PBS + 1% BSA overnight at 4 °C. Staining was completed the following day with the RTU secondary kits (Vector) applied for 1 h and then administration of 3, 3′-diaminobenzidine (DAB) (Vector) for up to 1 min. Terminal deoxynucleotidyl transferase dUTP nick end labeling (TUNEL) stainings were conducted with the TACS TdT-DAB In Situ Apoptosis Detection kit (R&D/Bio-Techne, Minneapolis, MN, USA) following manufacturer’s instructions with the following key details: proteinase K was incubated for 15 min and TdT labeling was done with the cobalt cation for 5 min. All slides were counterstained with hematoxylin and coverslipped with a Tissue-Tek Prisma Plus with Film automated slide stainer (Sakura Finetek, Torrance, CA, USA).

Histological quantifications were conducted blinded using 40x images in ImageJ (NIH) for baseline, control and virus exposed samples from all ZIKV (*n* = 10) and iZIKV (*n* = 9) perfusion experiments. Images were acquired with a Leica SP8 system and DFC7000 camera (Buffalo Grove, IL, USA). A minimum of 3 images from each section/staining were analyzed to generate a mean value for each biological sample. For syncytium Injury, the amount of Placental Alkaline Phosphatase (PLAP) staining within regions of interest drawn over damaged areas was measured relative to the total area of PLAP staining per field. The total area of PLAP staining constitutes the proportion of PLAP pixels over tissue pixels, omitting the intervillous space to account for differences in the amount of syncytium between fields. TUNEL or Ki67 positive nuclei were counted using the ImageJ cell counter application. CD163 was measured as the percentage of DAB staining within each field. All histological counts/measurements were normalized to the area of placental tissue (omitting the maternal space) within each respective image.

### Immunofluorescence (IF)

A second 1 cm x 1 cm biopsy was fixed in 4% formaldehyde in PBS (made from powder, Sigma) for 2 h and left overnight in PBS + 30% Sucrose. Samples were embedded in OCT and frozen with 2-methylbutane cooled below −150 °C using LN2. Sections were cut 8 µm thick and blocked once more overnight with PBS + 1% BSA with 10% Donkey serum. Primary antibodies were again diluted in PBS + 1% BSA and left overnight at 4 °C. Secondary antibodies were also diluted in PBS + 1% BSA and applied for 1 h. DAPI (Thermo) was applied before coverslipping with ProLong Diamond mounting media (Thermo). Confocal images were acquired using a DMi8 TCS SPE II system (Leica) using the same parameters for controls and experimental samples. Antibodies details and dilutions for IHC and IF are listed in Supplementary Table 2.

### Plaque assay

Vero cells were seeded into 12-well plates at 5 × 10^5^ cells per well. After 24 h, monolayers were inoculated with 100 µl of 10-fold serial dilution of ZIKV or iZIKV stock in DMEM supplemented with 2% FBS and 1% Antibiotic Antimycotic. The plates were rocked every 15 mins for 1 h at 37 °C after which inoculum was removed and replaced with a 1% agar overlay. Four days later a second 1% agarose overlay containing 2% Neutral Red (Sigma-Aldrich) was added for 4 h at which point plaques became visible. After visual confirmation of plaques, plates were fixed in 10% neutral buffered formalin for 1 h. After fixation agar plugs were removed and the plates were stained with 1% crystal violet for 5 min. Crystal violet was then removed and plates were air dried and imaged.

### RNA extraction

0.5 cm × 0.5 cm biopsies were placed in individual tubes containing 4 ml ice-cold RNAlater (Thermo) and transferred to 4 °C. Within 7 days, samples were transferred from the RNAlater to empty tubes and placed at −80 °C for long-term storage. At the time of sample processing, tissues were weighed and homogenized in 1 ml Qiazol Reagent (QIAGEN, Valencia, CA, USA) using a bead-beater apparatus (Precellys, Bertin Corp., Rockville, MD, USA). Total RNA was isolated from 350 µl homogenate using a phenol-chloroform extraction method and then purified with the RNeasy Lipid Tissue Mini Kit (QIAGEN, Valencia, CA, USA), in accordance with the manufacturer’s instruction. RNA concentration was measured on the Qubit Fluorometer (Thermo) using the Qubit RNA Broad-Range Assay Kit (Thermo).

### Viral load analysis

Viral RNA was measured in placenta tissue using a ZIKV prME-specific qRT-PCR assay. Three biopsies from each of the ten ZIKV exposed cotyledons were individually analyzed. A total of 400 ng of RNA was used to generate cDNA using the iScript Select cDNA Synthesis Kit (Bio-Rad, Hercules, CA, USA) following the manufacturer’s protocol for gene-specific priming. Viral RNA copy number was quantified using the TaqMan Universal Master Mix containing AmpliTaq Gold DNA Polymerase (Thermo) on a ViiA7 Real-time PCR System instrument. ZIKV primers and probe were previously described and correspond to residues in the ZIKV FSS13025 prME genomic region (GenBank no. MH368551) that are conserved in the ZIKV Brazil genome (GenBank no. KX811222**)**. Standard dilutions and experimental cDNAs were run in triplicate. The standard curve was used to calculate the ZIKV copy number per µg of tissue. Samples with at least two of three replicates within the linear range of the standard curve were considered positive. To adhere to stringent guidelines, Ct (cycle threshold) values >38 were deemed as not reliably detected and were not reported. Baseline and control-perfused for the ten placenta donors were determined to be negative based on these criteria. Copy number sensitivity using a standard curve from serial dilutions of ZIKV genome was 25 copies/qRT-PCR reaction.

### Gene expression analysis

The NanoString nCounter platform (NanoString, Seattle, WA, USA) as used to assess placenta gene expression in sixty tissue biopsies. Two biopsies per cotyledon were analyzed for each condition (Baseline, Control, ZIKV, and iZIKV) from *n* = 5 ZIKV and *n* = 5 iZIKV perfusion experiments. From each sample, 150 ng RNA was processed and loaded in accordance with manufacturer’s instructions for targeted expression with the NanoString Human PanCancer Immunology Panel. Following the acquisition, we normalized gene expression values by estimating factors of unwanted variation with RUVg^79^. This approach uses the NanoString panel of housekeeping genes to estimate factors of technical variation observed in the dataset^79^. We selected one factor of unwanted variation to remove from the dataset and visualized RUVg normalized counts via principal component analysis and relative log expression plots, which resulted in the removal of a single outlier. Using the raw count matrix with the outliers removed, we performed trimmed mean of M-values normalization with edgeR and logCPM transformation with the voom function in limma^80–83^. The normalized count matrix was fit to a linear model with treatment included as the main effect and the RUVg factor of unwanted variation included as a covariate. Differential expression testing was performed in limma using a robust empirical Bayes moderation of the fitted linear model to control for hypo- & hyper-variable genes (e.g. sex-specific gene expression)^84^. Paired differential expression contrasts were performed for each treatment group against their matched controls. A difference of differences contrast (i.e. interaction model) was also performed to compare the responses to iZIKV exposure and ZIKV infection while controlling for baseline differences. In each contrast, a gene was considered DE if the unadjusted *p* value < 0.05, as suggested by the NanoString Gene Expression Data Analysis Guidelines. The union of DE genes in at least one contrast were visualized in a heatmap using the R package pheatmap.

### *IFITM1* Quantitative Real-Time PCR

This analysis was conducted on the same samples examined with the NanoString nCounter platform. Coding DNAs were generated from 100 ng total RNA using random priming that followed the iScript™ cDNA Synthesis Kit (Bio-Rad) instructions. The PCR was performed using the SYBR Green-Real-Time PCR Master Mix (Thermo) and with gene primer sets human *IFITM1* (Interferon Inducted Transmembrane Protein 1) and human *RPL13A* (Ribosomal Protein L13a), previously reported^58^. Plates were run on a ViiA 7 Real-Time PCR System with 384-well block (Thermo). Reactions for *hIFITM1* and *hRPL13A* were done in triplicate and the relative expression was calculated by the delta-delta Ct method.

### Statistics and Reproducibility

Datasets for Figs. 1–8 and supplementary data 1 were analyzed using Excel (Microsoft, Redmond, WA, USA) and SPSS (IBM, Armonk, NY, USA). Quantitative comparisons were conducted with paired or unpaired student’s *t*-test, as appropriate, and one-way ANOVA with Tukey’s post hoc test. Categorical data was compared with chi-squared test. Experiments comparing ZIKV were reproduced across 10 placentas. For experiments involving iZIKV, 9 placentas were utilized.

### Bioinformatics analysis

NanoString gene expression analysis was conducted in RStudio version 1.2.1335 with R version 4.0.0 using the following Bioconductor version 3.11 libraries: RUVSeq version 0.99.1, limma version 3.44.1, edgeR version 3.30.0, and EDASeq version 2.22.0^85^. Functional analysis of gene expression datasets was performed using Ingenuity^®^ Pathway Analysis (IPA, QIAGEN, Inc.)^86^. The software tool analyzes the experimental dataset in the context of known biological functions and pathways within the Ingenuity Pathways Knowledge Base, a curated repository of biological interactions and functional annotations. Canonical pathways analysis identifies IPA pathways that were significantly enriched (*p* < 0.05) and predicts the changes in pathways based on the observe expression changes of genes within each pathway. The *p*-value associated with each pathway was calculated using the right-tailed Fisher’s exact test.

### Figures

Graphs were generated using Excel, PowerPoint (Microsoft), and R. Figures were compiled using Photoshop (Adobe, San Jose, CA, USA). Brightness and contrast were adjusted as necessary to increase visibility; controls were adjusted equally to experimental samples. NanoString figures were generated with the R graphics package for volcano plots and pheatmap (version 1.0.123) for co-expression heatmaps.

### Reporting summary

Further information on research design is available in the Nature Research Reporting Summary linked to this article.

## Supplementary information

Supplementary Information

Description of Additional Supplementary Files

Supplementary Data 1

Supplementary Data 2

Reporting summary

## Data Availability

The source data behind the graphs are available in Supplementary Data 2. All other data are available from the corresponding author on reasonable request and with institutional approval.
