# Supplementary material for: ZIKV can infect human term placentas in the absence of maternal factors
Source: Commun Biol. 2022 Mar 18;5:243. doi: 10.1038/s42003-022-03158-6 (PMC8933440; doi:10.1038/s42003-022-03158-6)
Supplement: Supplementary file 1 — Supplementary Information [file 42003_2022_3158_MOESM1_ESM.pdf]

ZIKV perfusion experiments

	Media	Control	ZIKV	Media vs. Control	Media vs. ZIKV	Control vs. ZIKV
**pH**	7.21 ±0.03	7.18 ±0.06	7.24 ±0.12	0.612	0.761	0.646
**PCO2 (mmHg)**	11.8 ±0.8	12.0 ±0.7	11.1 ±1.1	0.864	0.594	0.738
**PO2 (mmHg)**	184.2 ±2.7	158.8 ±3.1	153.6 ±3.3	***$4.23x10^{-05}$***	***$5.39x10^{-06}$***	0.262
**Lactate (mmol/L)**	<0.30 ±0.00	0.41 ±0.06	0.44 ±0.08	0.118	0.120	0.733
**HCO3 (mmol/L)**	4.63 ±0.34	4.43 ±0.41	5.36 ±1.21	0.641	0.494	0.476
**Mean ±SEM**				**p-value paired *t*-test**		**p-value unpaired *t*-test**

iZIKV perfusion experiments

	Media	Control	iZIKV	Media vs. Control	Media vs. iZIKV	Control vs. iZIKV
**pH**	7.13 ±0.02	7.08 ±0.04	7.16 ±0.09	0.380	0.759	0.527
**PCO2 (mmHg)**	10.7 ±0.5	11.22 ±1.1	10.5 ±1.1	0.810	0.816	0.650
**PO2 (mmHg)**	190.9 ±3.5	172.3 ±4.8	172.5 ±4.2	***0.010***	***0.007***	0.980
**Lactate (mmol/L)**	<0.30 ±0.00	0.30 ±0.00	0.38 ±0.06	0.363	0.220	0.290
**HCO3 (mmol/L)**	3.48 ±0.10	3.28 ±0.09	3.25 ±0.13	0.064	0.224	0.847
**Mean ±SEM**				**p-value paired *t*-test**		**p-value unpaired *t*-test**

Supplementary Table 1: Venous effluent metabolic analysis. Metabolic analysis of the media at baseline (denoted as media) before perfusion into the artery and following efflux from the vein (denoted as venous effluent) collected at 28 hours from control and virus exposed cotyledons. Values in the left columns reflect averages and ± standard error of the mean (SEM) for respective parameters. In the columns to the right, p-values <0.05 are in italic and bold font.

Supplementary Table 2. Antibodies

Name/target	Type	Clone	Species	Manufacturer	Catalog Number	Dilution used 1:	Final Concentration
NS1	IgG	EA88	Mouse	Thermo	MA5-24583	50	2 µg/ml
CD163	IgG	EPR19518	Rabbit	Abcam	ab182422	500	1.39ug/ml
Cytokeratin 7	IgG	EPR1619Y	Rabbit	Abcam	ab68459	1000	0.11 µg/ml
PLAP	IgG	EPR6141	Rabbit	Abcam	ab133602	500	0.19 µg/ml
Isotype to NS1	IgG	P3.6.2.8.1	Mouse	eBiosceince-Thermo	12-4714-42	250	2 µg/ml
Isotype to others	IgG	EPR25A	Rabbit	Abcam	ab172730	10000	1.34ug/ml
Ki67	IgG	Polyclonal	Rabbit	Abcam	ab15580	1000	0.9 µg/ml
WGA	Lectin	N/A	N/A	Vector Labs	B-1025	500	2 µg/ml
**Secondaries**	**Type**	**Clone**	**Species**	**Manufacturer**	**Catalog Number**	**Dilution used 1:**	**Final Concentration**
Anti-Mouse Alexa 555	IgG	Polyclonal	Donkey	Invitrogen-Thermo	A31570	1000	2 µg/ml
Anti-Rabbit Alexa 488	IgG	Polyclonal	Donkey	Jackson Labs	711-546-152	1000	1.5ug/ml
Anti-Ms RTU	IgG	Polyclonal	Horse	Vector Labs	BP-2000	used as recommended	n/a
Anti-Rb RTU	IgG	Polyclonal	Horse	Vector Labs	BP-1100	used as recommended	n/a
Streptavidin Alexa 647	N/A	N/A	N/A	Jackson Labs	016-600-084	1000	1.8 µg/ml
