# Supplementary material for: ZIKV can infect human term placentas in the absence of maternal factors
Source: Commun Biol. 2022 Mar 18;5:243. doi: 10.1038/s42003-022-03158-6 (PMC8933440; doi:10.1038/s42003-022-03158-6)
Supplement: Supplementary file 2 — Description of Additional Supplementary Files [file 42003_2022_3158_MOESM2_ESM.pdf]

Description of Additional Supplementary Files

File name: Supplementary Data 1
Description: Placenta Donor Characteristics.

File name: Supplementary Data 2
Description: Source data behind the figures.
