# Supplementary material for: ZIKV can infect human term placentas in the absence of maternal factors
Source: Commun Biol. 2022 Mar 18;5:243. doi: 10.1038/s42003-022-03158-6 (PMC8933440; doi:10.1038/s42003-022-03158-6)
Supplement: Supplementary file 3 — Supplementary Data 1 [file 42003_2022_3158_MOESM3_ESM.docx]

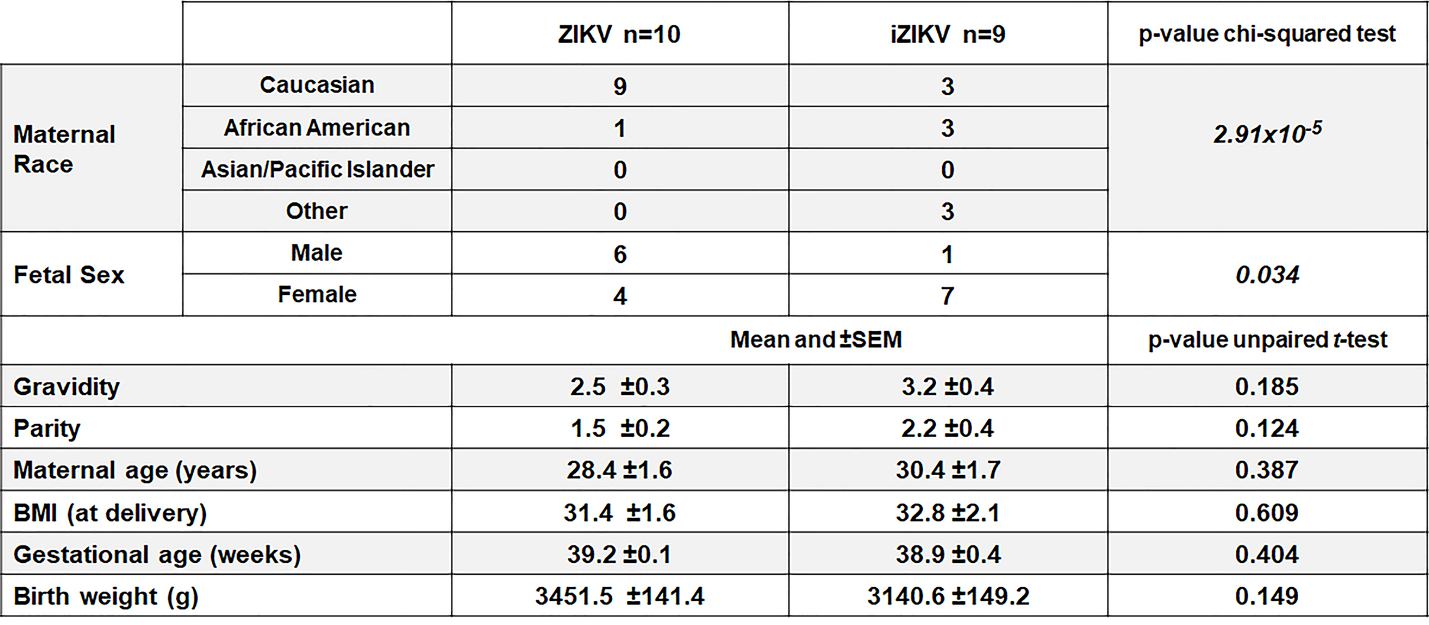


**Supplementary Data 1: Placenta Donor Characteristics.** Maternal and fetal characteristics for placentas perfused with ZIKV and iZIKV, respectively. As indicated, p-values between experimental groups were generated by chi-squared or student’s *t*-test. SEM denotes the standard error of the mean.
