# Supplementary material for: ZIKV can infect human term placentas in the absence of maternal factors
Source: Commun Biol. 2022 Mar 18;5:243. doi: 10.1038/s42003-022-03158-6 (PMC8933440; doi:10.1038/s42003-022-03158-6)
Supplement: Supplementary file 5 — Reporting summary [file 42003_2022_3158_MOESM5_ESM.pdf]

nature portfolio

Corresponding author(s): Nicholas Ieronimakis, Michael Gale Jr.

Last updated by author(s): Nov 3, 2021

Reporting Summary

Nature Portfolio wishes to improve the reproducibility of the work that we publish. This form provides structure for consistency and transparency in reporting. For further information on Nature Portfolio policies, see our Editorial Policies and the Editorial Policy Checklist.

Statistics

For all statistical analyses, confirm that the following items are present in the figure legend, table legend, main text, or Methods section.

n/a	Confirmed	
☐	☒	The exact sample size (*n*) for each experimental group/condition, given as a discrete number and unit of measurement
☐	☒	A statement on whether measurements were taken from distinct samples or whether the same sample was measured repeatedly
☐	☒	The statistical test(s) used AND whether they are one- or two-sided<br>*Only common tests should be described solely by name; describe more complex techniques in the Methods section.*
☐	☒	A description of all covariates tested
☐	☒	A description of any assumptions or corrections, such as tests of normality and adjustment for multiple comparisons
☐	☒	A full description of the statistical parameters including central tendency (e.g. means) or other basic estimates (e.g. regression coefficient) AND variation (e.g. standard deviation) or associated estimates of uncertainty (e.g. confidence intervals)
☐	☒	For null hypothesis testing, the test statistic (e.g. *F*, *t*, *r*) with confidence intervals, effect sizes, degrees of freedom and *P* value noted<br>*Give P values as exact values whenever suitable.*
☒	☐	For Bayesian analysis, information on the choice of priors and Markov chain Monte Carlo settings
☒	☐	For hierarchical and complex designs, identification of the appropriate level for tests and full reporting of outcomes
☒	☐	Estimates of effect sizes (e.g. Cohen's *d*, Pearson's *r*), indicating how they were calculated

Our web collection on statistics for biologists contains articles on many of the points above.

Software and code

Policy information about availability of computer code

Data collection	No software was used.
Data analysis	RStudio version 1.2.1335 with R version 4.0.0 using the following Bioconductor version 3.11 libraries: RUVSeq version 0.99.1, limma version 3.44.1, edgeR version 3.30.0, aEDASeq version 2.22.0 and Ingenuity® Pathway Analysis (IPA, QIAGEN, Inc.).

For manuscripts utilizing custom algorithms or software that are central to the research but not yet described in published literature, software must be made available to editors and reviewers. We strongly encourage code deposition in a community repository (e.g. GitHub). See the Nature Portfolio guidelines for submitting code & software for further information.

Data

Policy information about availability of data

All manuscripts must include a data availability statement. This statement should provide the following information, where applicable:

- Accession codes, unique identifiers, or web links for publicly available datasets
- A description of any restrictions on data availability
- For clinical datasets or third party data, please ensure that the statement adheres to our policy

Raw data will be made available upon request from interested parties.

Field-specific reporting

Please select the one below that is the best fit for your research. If you are not sure, read the appropriate sections before making your selection.

☒ Life sciences ☐ Behavioural & social sciences ☐ Ecological, evolutionary & environmental sciences

For a reference copy of the document with all sections, see nature.com/documents/nr-reporting-summary-flat.pdf

Life sciences study design

All studies must disclose on these points even when the disclosure is negative.

Sample size	No sample power analysis was possible to determine human placenta numbers as this has never been successfully reported for virus perfusion's. Based on our previous studies with placenta perfusion we reasoned upwards of ten would be suitable.
Data exclusions	No exclusions.
Replication	The human dual cotyledon, dual perfusion assay is unique and not widely available. We welcome reproduction by other groups and hope publication will draw attention to the utility of this assay towards understanding human placental infection.
Randomization	Cotyledons were randomized based on a random numbering table.
Blinding	Data acquisition was conducted blinded relative to the treatment of each cotyledon/sample.

Reporting for specific materials, systems and methods

We require information from authors about some types of materials, experimental systems and methods used in many studies. Here, indicate whether each material, system or method listed is relevant to your study. If you are not sure if a list item applies to your research, read the appropriate section before selecting a response.

Materials & experimental systems

n/a	Involved in the study	
☐	☒	Antibodies
☒	☐	Eukaryotic cell lines
☒	☐	Palaeontology and archaeology
☒	☐	Animals and other organisms
☐	☒	Human research participants
☐	☒	Clinical data
☒	☐	Dual use research of concern

Methods

n/a	Involved in the study	
☒	☐	ChIP-seq
☒	☐	Flow cytometry
☒	☐	MRI-based neuroimaging

Antibodies

Antibodies used	Antibodies details including suppliers are listed in the supplementary file 1.
Validation	We utilized positive and negative controls as well as IgG isotypes to validate certain antibodies. Some are also well validated and/or published by suppliers.

Human research participants

Policy information about studies involving human research participants

Population characteristics	Clinical characteristics for placentas used are provided in supplementary data 1.
Recruitment	Donors were recruited prior to their scheduled cesareans only if their pregnancies were uncomplicated. Pregnancies with complications, tobacco, drug or alcohol consumption (if known) were excluded from recruitment.
Ethics oversight	This study was approved by the Madigan Army Medical Center IRB, where placentas were collected and perfusion conducted.

Note that full information on the approval of the study protocol must also be provided in the manuscript.

Clinical data

Policy information about clinical studies

All manuscripts should comply with the ICMJE guidelines for publication of clinical research and a completed CONSORT checklist must be included with all submissions.

Clinical trial registration	not applicable
Study protocol	The IRB protocol number is 217032
Data collection	Clinical characteristics were collected by clinical staff via accessing the medical record in accordance with patients consent.
Outcomes	Our main outcomes were Zika virus infection and placental immune responses in our ex vivo perfusion model.
